# Pathways and Mechanisms of Cellular Cholesterol Efflux—Insight From Imaging

**DOI:** 10.3389/fcell.2022.834408

**Published:** 2022-03-01

**Authors:** Alice Dupont Juhl, Daniel Wüstner

**Affiliations:** Department of Biochemistry and Molecular Biology, PhyLife, Physical Life Sciences, University of Southern Denmark, Odense, Denmark

**Keywords:** cholesterol, oxysterol, HDL, LDL, ABC transporter, extracellular vesicles, niemann pick disease, apoprotein A1 (Apo A1)

## Abstract

Cholesterol is an essential molecule in cellular membranes, but too much cholesterol can be toxic. Therefore, mammalian cells have developed complex mechanisms to remove excess cholesterol. In this review article, we discuss what is known about such efflux pathways including a discussion of reverse cholesterol transport and formation of high-density lipoprotein, the function of ABC transporters and other sterol efflux proteins, and we highlight their role in human diseases. Attention is paid to the biophysical principles governing efflux of sterols from cells. We also discuss recent evidence for cholesterol efflux by the release of exosomes, microvesicles, and migrasomes. The role of the endo-lysosomal network, lipophagy, and selected lysosomal transporters, such as Niemann Pick type C proteins in cholesterol export from cells is elucidated. Since oxysterols are important regulators of cellular cholesterol efflux, their formation, trafficking, and secretion are described briefly. In addition to discussing results obtained with traditional biochemical methods, focus is on studies that use established and novel bioimaging approaches to obtain insight into cholesterol efflux pathways, including fluorescence and electron microscopy, atomic force microscopy, X-ray tomography as well as mass spectrometry imaging.

## 1 Cholesterol–An Import Lipid Constituent of Cellular Membranes

Cholesterol is a small lipid molecule consisting of a steroid ring system, a short alkyl-chain and a hydroxy group at position 3 as the only polar constituent. These properties, together with the asymmetric orientation of its methyl groups, give cholesterol a unique ability to interact with phospholipids in cellular membranes. Cholesterol reduces the propensity of gauche configurations in carbon chains, thereby ordering the fatty acid acyl chains, decreasing the distance between phospholipid head groups and condensing the lipid bilayer (Mouritsen and Zuckermann, 2004). This ability critically depends on both, the steroid back bone and the aliphatic side chain of cholesterol (Ipsen et al., 1990; Henriksen et al., 2006; Scheidt et al., 2013). As a result of this interaction, membrane permeability and bending flexibility are reduced, while lateral lipid diffusion is largely preserved (Ipsen et al., 1990). Thus, cholesterol has the ability to maintain lipid and protein mobility in a membrane, while controlling membrane thickness and flexibility as well as the membrane barrier function to metabolites, ions, and signaling molecules. Its presence creates free energy penalties for conformational transitions of proteins in cholesterol-containing membranes, e.g., due to hydrophobic mismatch or bending resistance in curved membrane regions and during vesicle formation (Lundbæck and Andersen, 2012). Energy barriers to be overcome by proteins during their catalytic cycle in membranes containing cholesterol compared to cholesterol-free membranes can be as high as 45 kJ/mol. This corresponds to 18 times the thermal energy and almost as much as hydrolysis of one ATP molecule (54 kJ/mol) (Lundbæck and Andersen, 2012). In addition, cholesterol can specifically interact with membrane proteins, thereby regulating their function. Prominent examples for regulation of protein function by cholesterol as allosteric ligand are G-protein coupled receptors or ligand-gated ion channels (de Almeida et al., 2004; Chattopadhyay et al., 2005). Cholesterol can also directly control signaling cascades, as shown by its specific binding to components of the Hedgehog pathway, such as Smoothened or Patched (Huang et al., 2016; Gong et al., 2018). Finally, cholesterol can be covalently attached to signaling proteins of the Hedgehog pathway, i.e., as a membrane anchor for Hedgehog and as a sensor to locate Smoothened to primary cilia (Garcia et al., 2001; Xiao et al., 2017). These diverse functions of cholesterol necessitate a tight control of its abundance in cellular membranes. We will start this review by introducing, how cholesterol synthesis and uptake are regulated before discussing mechanisms of cellular efflux of excess cholesterol.

## 2 Cholesterol Homeostasis by Feedback-Regulated *de novo* Synthesis and Cellular Uptake

### 2.1 A Short Detour Into Cholesterol Synthesis

All carbon atoms in cholesterol originate from acetate, and cholesterol-specific biosynthesis starts with the reduction of the activated ketone body hydroxymethylglutaryl-Coenzyme A into mevalonate by the cytoplasmic enzyme hydroxymethylglutaryl-Coenzyme A reductase (HMG-CoA reductase) (Bloch, 1965). This is followed by a sequential synthesis of five-carbon isoprene units, which can condense to form geranyl- and from there farnesyl-pyrophosphate (a 15-carbon isoprene), which condenses in a head-to-tail-manner to form squalene. Upon oxygen-dependent epoxide formation to activate squalene, cyclization into the steroid backbone to form lanosterol is catalyzed by oxidosqualene cyclase. From lanosterol to cholesterol, either the Bloch or the Kandutsch-Russel pathway are used, which involve an additional 8-9 reaction steps to finally form cholesterol (Cerqueira et al., 2016). Thus, the biosynthesis of cholesterol is very complex, leading also to other important biomolecules, which are either needed in oxidative phosphorylation, such as ubiquinone, for glycosylation reactions, for example, dolichol, or for hydrophobic anchoring of peripheral membrane proteins (e.g. via farnesylations) (Cerqueira et al., 2016). Statins, which are used to lower blood cholesterol levels for the prevention of atherosclerosis, are competitive inhibitors of HMG-CoA reductase, resulting not only in inhibition of synthesis of cholesterol but also of such important isoprene derivatives (Cerqueira et al., 2016).

### 2.2 Cellular Uptake and Intracellular Fate of Low Density Lipoprotein

Low density lipoprotein (LDL) provides cholesterol for uptake into cells. Upon binding to the LDL-receptor at the plasma membrane (PM), the LDL/LDL receptor complex can be internalized by clathrin-dependent endocytosis (Iaea and Maxfield, 2015; Pfisterer et al., 2016). Adaptor proteins, such as autosomal recessive hypercholesterolemia (ARH) protein are essential for endocytosis of the LDL/LDL receptor complex, and mutations in ARH or the LDL receptor can lead to inherited forms of hypercholesterolemia (Garcia et al., 2001). Upon endocytosis, newly formed endosomes, containing both LDL and its receptor, will release the clathrin coat and start to fuse with pre-existing sorting/early endosomes located in the cellular periphery. Due to a slight drop in pH in the sorting endosomes compared to the cell surface, the LDL-receptor undergoes a conformational change and releases the LDL particle. The free LDL-receptor will subsequently recycle back to the cell surface, either directly from sorting endosomes or via the endocytic recycling compartment (ERC) (Maxfield and Mcgraw, 2004). Exit of the LDL receptor from the sorting endosomes takes place primarily by narrow diameter tubules ensuring a high surface-to-volume ratio for efficient membrane recycling. The released LDL is retained in the lumen of sorting endosomes, which slowly mature into late endosomes, thereby losing their fusion capacity for incoming vesicles and acquiring acid hydrolases from the trans-Golgi network (TGN) for degradation of luminal cargo (Maxfield and Mcgraw, 2004). The LDL particles will be degraded in late endosome and lysosomes (LE/LYSs) where LDL-associated cholesteryl esters (CEs) will be hydrolyzed by acid lipase to unesterified (free) cholesterol and free fatty acids (Chao et al., 1992; Ameis et al., 1994). Some of the liberated cholesterol can be re-esterified by acyl-Coenzyme A acyl transferase (ACAT) in the endoplasmic reticulum (ER) and stored as CEs in lipid droplets (LDs).

### 2.3 Regulation of Cholesterol Synthesis and Uptake

**FIGURE 1:**
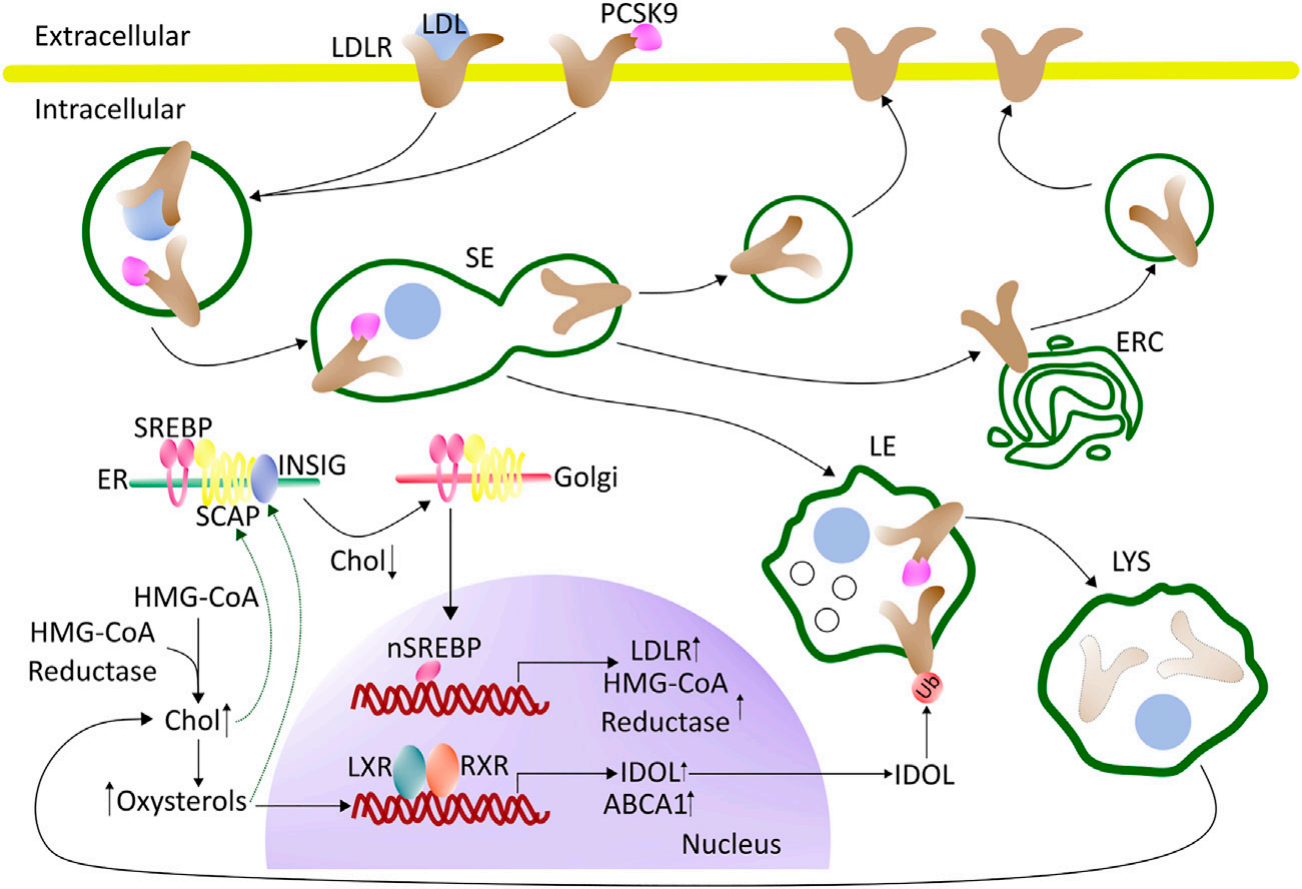
Regulation of cholesterol synthesis and uptake - an overview. LDL binds to the LDL receptor (LDLR) at the cell surface and becomes internalized by clathrin-mediated endocytosis. Upon arrival in the sorting endosomes (SE), LDL dissociates from the LDLR and is retained in the lumen of SE which gradually mature into late endosomes (LE). The LDLR recycles back to the cell surface, either directly from the SE or by passing through the endocytic recycling compartment (ERC). The maturation process of SE into LE, and the further conversion of LE into lysosomes (LYS), involves the formation of internal vesicles in LE, acquisition of LBPA, a drop in pH, and import of proteases, lipases, and other lysosomal enzymes from the Golgi. When the cell is rich in cholesterol, SREBP will be retained in the ER by SCAP and Insig. Cholesterol will bind to SCAP, whereas oxysterol will bind to Insig (green arrows). LXR will be activated by oxysterols, which together with RXR can induce the transcription of genes involved in protecting the cells from becoming overloaded with cholesterol. During low cholesterol conditions, the SCAP-SREBP complex will be released from Insig in the ER and move to the Golgi for cleavage and activation (nSREBP). In the nucleus, nSREBP can activate the transcription of genes involved in cholesterol biosynthesis and uptake. The expression of PCSK9, which promotes the degradation of the LDL-receptor by interfering with its recycling, is also under the control of the nSREBP system. Intracellular degradation of LDLR is promoted by the E3 ubiquitin ligase IDOL, whose expression is stimulated by LXR under conditions of high cellular cholesterol. See the text for more details. ABAC1 (ATP-binding cassette A1), Chol (cholesterol), ER (endoplasmic reticulum), ERC (endocytic recycling compartment), HMG-CoA (3-hydroxy-3-methylglutaryl-Coenzyme A), IDOL (inducible degrader of the LDL receptor), LDL (low-density lipoprotein), LDLR (LDL-receptor), LXR (liver-X receptor), PCSK9 (proprotein convertase substilisin/kexin type 9), RXR (retinoid X receptor) SCAP (SREBP-Cleavage-Activating protein), SE (sorting endosomes), SREBP (sterol regulatory element-binding protein), Ub (ubiquitin).

Since several important regulators of cholesterol homeostasis reside in the ER and the sterol concentration in this organelle is low under physiological conditions, the ER is an excellent control center for cholesterol homeostasis. Cells respond to a drop in cholesterol levels with increased expression of HMG-CoA reductase and other biosynthetic enzymes, which is an important feedback mechanism. (Istvan and Deisenhofer, 2001). This feedback is based on the ER-resident sterol regulatory binding protein (SREBP). SREBP is retained in the ER together with the cholesterol-sensing protein (SCAP), and the two ER retention proteins Insig-1 or -2, during cholesterol replete conditions (Iaea and Maxfield, 2015). Under such condition, cholesterol will bind to SCAP, causing it to bind to Insig (Adams et al., 2004), while oxysterols will bind to Insig promoting it to bind and retain SCAP in the ER (Radhakrishnan et al., 2007; Luo et al., 2020). Once the ER cholesterol level drops below 5 mol% SCAP will undergo a conformational change that will cause the SCAP-SREBP complex to be released from Insig (Radhakrishnan et al., 2008). The SCAP-SREBP complex can then be transferred in COPII coated vesicles to the Golgi apparatus, where SREBP will be activated by cleavage and release of its N-terminal transcription factor (nSREBP). In the nucleus, nSREBP can activate the transcription of genes involved in cholesterol metabolism, synthesis, and uptake such as the HMG-CoA reductase and the LDL-receptor (Horton et al., 2003; Yang et al., 2020). Remarkably, nSREBP controls both LDL endocytosis, by activating the transcription of LDL-receptor, and at the same time down-regulation of the same receptor by inducing the transcription of the Proprotein convertase subtilisin/kexin type 9 (PCSK9; Figure 1). PCSK9 is a secreted protein, which binds to the extracellular site of LDL-receptor on the PM and becomes internalized together with the LDL-receptor. Once inside early endosomes, PCSK9 prevents the pH-dependent conformational change of the LDL-receptor, which is necessary for its recycling, promoting receptor degradation in the LE/LYSs (McNutt et al., 2007; Mousavi et al., 2009; Zhang et al., 2012). Upon nSREBP-stimulated synthesis, LDL receptor can also be escorted by PCSK on the secretory pathway from the TGN directly to LE/LYSs for degradation (Schilling et al., 2004). This complex control mechanism of LDL receptor synthesis and degradation by sterol-induced regulation of nSREBP activity shows the importance of tight surveillance of LDL uptake into cells for overall cholesterol homeostasis.

In addition to the SREBP/SCAP/Insig system, nuclear receptors play a major role in cholesterol regulation. For example, the liver X receptors (LXRs) function as complementary and independent sterols sensors, which are particularly important for the control of cellular cholesterol efflux. LXRs are activated by oxysterols, which are generated when cellular cholesterol levels are high (Luu et al., 2016). Upon activation LXRs together with isomers of retinoid X receptors (RXRs) will bind as heterodimers to their DNA response element (Figure 1) (Zhao and Dahlman-Wright, 2010). This will induce the transcription of genes that are involved in protecting the cell from becoming overloaded with cholesterol, including the ATP-binding cassette transporter A1 (ABCA1) which mediates the egress of phospholipids and cholesterol to acceptor proteins, such as apolipoprotein A-1 (apoA1) (Venkateswaran et al., 2000). To prevent a futile cycle of cellular cholesterol uptake via endocytosis of LDL and cholesterol efflux by ABC transporters, the inducible degrader of LDL-receptor (IDOL), an E3 ubiquitin ligase, is also regulated by the LXR pathway (Zhang et al., 2012; Scotti et al., 2013). The function of IDOL is to ubiquitinate the LDL-receptor, which leads to its association with components of the endosomal sorting complex required for transport (ESCRT) (Scotti et al., 2013). As a consequence, the LDL-receptor is retained in LE/LYSs and degraded. ESCRT is not only essential for the degradation of endosomal cargo but also for the formation of cholesterol-rich intraluminal vesicles (ILVs), which accumulate in late endosomes, as they mature (Gruenberg, 2020). By ubiquitinating the LDL receptor and thereby stimulating its lysosomal degradation, IDOL limits the uptake of cholesterol when cellular cholesterol levels are high (Zelcer et al., 2009; Scotti et al., 2011; Zhang et al., 2012) (Figure 1). How precisely IDOL mediates degradation of the LDL receptor is not known, but it seems to be unaffected by proteasome blockers, suggesting that it takes place in endo-lysosomes (Zelcer et al., 2009). Additional regulatory mechanisms for LDL-receptor recycling versus degradation have been discovered and are discussed in a recent dedicated review (Vos and van de Sluis, 2021). Ubiquitination followed by proteasomal degradation is another important regulatory mechanism, which controls the abundance of SREBPs, LXRs, the LDL-receptor, and ATP-binding cassette (ABC) transporters, such as ABCA1 (Sharpe et al., 2014). The ubiquitin proteasome system (UPS) also limits the abundance of enzymes for cholesterol biosynthesis, thereby controlling the overall flux through this pathway (Heinrich and Schuster, 1996; Sharpe et al., 2014).

## 3 Cholesterol Distribution in Cells and the Chemical Potential of Cholesterol in Cellular Membranes

### 3.1 Cholesterol Distribution Between Subcellular Membranes

Cholesterol is most abundant in the PM, the endocytic pathway, and the TGN with lower concentrations in the ER, mitochondria, and other organelles. Since cholesterol can exchange between organelle membranes by non-vesicular transport without apparent free energy consumption, a homogeneous distribution would be expected, if the chemical potential of cholesterol in all membranes would be equal (Maxfield and Menon, 2006; Wüstner and Solanko, 2015). To prevent that, cells seem to use the free energy gained from ATP-hydrolysis to generate different phospho- and sphingolipid compositions of subcellular organelles. This creates characteristic conditions for the specific interaction of cholesterol in each organelle, which, in turn, indirectly affects the distribution of cholesterol between cellular membranes. Lipid gradients could be maintained by active phospholipid transport, for example via ABC transporters or by enzyme-catalyzed phospholipid modifications, e.g., phosphorylation and dephosphorylation of phosphatidyl inositol species at the ER-Golgi interface (Mesmin and Antonny, 2016). As a consequence, the chemical potential of cholesterol between different organelle membranes could be the same, despite quite different concentrations.

### 3.2 Active Cholesterol as a Useful Thermodynamic Concept to Rationalize Non-vesicular Transport

Biophysical studies in binary and ternary model membranes have shown that cholesterol can cause the formation of a liquid-ordered phase coexisting with a fluid lipid phase at physiologically relevant temperatures (Ipsen et al., 1987). Above a critical sterol mole fraction, cholesterol can also precipitate from membranes as aggregates and even form cholesterol monohydrate crystals. This pure cholesterol phase forms when the capacity of phospholipids to interact with and thereby solubilize cholesterol in the bilayer is exceeded (Huang. et al., 1999; Bach and Wachtel, 2003). Thus, the chemical potential of cholesterol is assumed to rise abruptly beyond this threshold concentration, leading to free cholesterol, which is not bound to phospholipids (Radhakrishnan and McConnell, 2000). This pool is often popularized as “active cholesterol” and assumed to play an important role in determining inter-organelle sterol fluxes for feedback regulation (Steck and Lange, 2010). For example, if the cholesterol concentration in the PM is raised beyond its physiological set point, non-vesicular transport of this excess cholesterol termed “active cholesterol” back to the ER could reestablish a steady state by shutting off cholesterol synthesis and LDL-mediated uptake via inhibition of the aforementioned SREBP/SCAP/Insig receptor system (Radhakrishnan et al., 2008; Sokolov and Radhakrishnan, 2010; Das et al., 2014). Since membrane cholesterol beyond a critical concentration is often detected using fluorescence labeled bacterial toxin derivatives, such as perifringolysin O (PFO), “active” cholesterol is sometimes also termed “accessible cholesterol” (Das et al., 2013). Concentration-dependent non-vesicular cholesterol transport from the PM to the ER and other organelles, such as LDs, could be mediated by the recently discovered GramD1/Aster proteins (Sandhu et al., 2018; Ferrari et al., 2020; Ercan et al., 2021), by oxysterol binding protein related protein 2 (ORP2) (Wang et al., 2019) or the steroid acute regulatory protein D4 (StARD4) (Mesmin et al., 2011; Iaea et al., 2020). Most of such lipid transfer proteins function at membrane contact sites, where membranes are tethered in close proximity, usually in the range of 5–50 nm, but never fully fuse (Scorrano et al., 2019). The reason for that could be to ensure increased efficiency of cholesterol flux between membranes when cholesterol’s chemical potential changes abruptly, since for large changes of the chemical potential, the flux-force relationship between the inter-membrane cholesterol flux and the chemical potential difference can become non-linear (Wüstner and Solanko, 2015). Thus, membrane contact sites might be necessary to enable rapid non-vesicular sterol fluxes when the cholesterol gradient between organelles increases beyond a critical set point.

### 3.3 Specific Interactions of Cholesterol With Phospholipids Might Determine Its Distribution in Cells

A variety of mechanisms including the formation of stoichiometric complexes, specific hydrogen bonding and lipid-specific shielding of cholesterol underneath phospholipid headgroups have been invoked to explain specific interactions of cholesterol with other lipids in cellular membranes (Huang. and Feigenson, 1999; Ohvo-Rekila et al., 2002; McConnell and Radhakrishnan, 2003). Notably, fluorescent analogues of cholesterol which have been added to cells in trace amounts or in exchange for some cholesterol have a homogeneous lateral distribution in the PM at least down to an accessible scale of 80 nm and diffuse faster than comparable phospholipid analogues (Wüstner et al., 2016; Pinkwart et al., 2019). These observations indicate that stoichiometric complexes of cholesterol with phospholipids would form only transiently in the PM. On the other hand, phospholipid-specific affinity of cholesterol could not only determine its distribution between cellular membranes at steady state (Wüstner and Solanko, 2015), but also dictate the transbilayer orientation and dynamics of cholesterol in the PM and other membranes. Both, preferred interactions of cholesterol with sphingolipids, like sphingomyelin (SM) and with phosphatidylserine (PS) compared to phosphatidylcholine (PC) or phosphatidylethanolamine (PE) have been described (Niu and Litman, 2002; Ohvo-Rekila et al., 2002; Nyholm et al., 2019). Recent studies in yeast and mammalian cells indicate that the majority of ergosterol and cholesterol reside in the cytoplasmic leaflet of the PM, which is rich in PS but not sphingolipids (Mondal et al., 2009; Maekawa and Fairn, 2015; Courtney et al., 2017; Solanko et al., 2018). Still, in both yeast and mammalian cells, sphingolipids, and PS asymmetry control sterol enrichment in the inner leaflet (Maekawa and Fairn, 2015; Courtney K. C. et al., 2018; Solanko et al., 2018). We will discuss the implications of these observations in the context of cholesterol efflux mechanisms from the PM below.

## 4 Cellular Efflux of Cholesterol and Formation of High-Density Lipoproteins

### 4.1 Cell-type Dependent Efflux of Cholesterol and Formation of High-Density Lipoprotein

**FIGURE 2:**
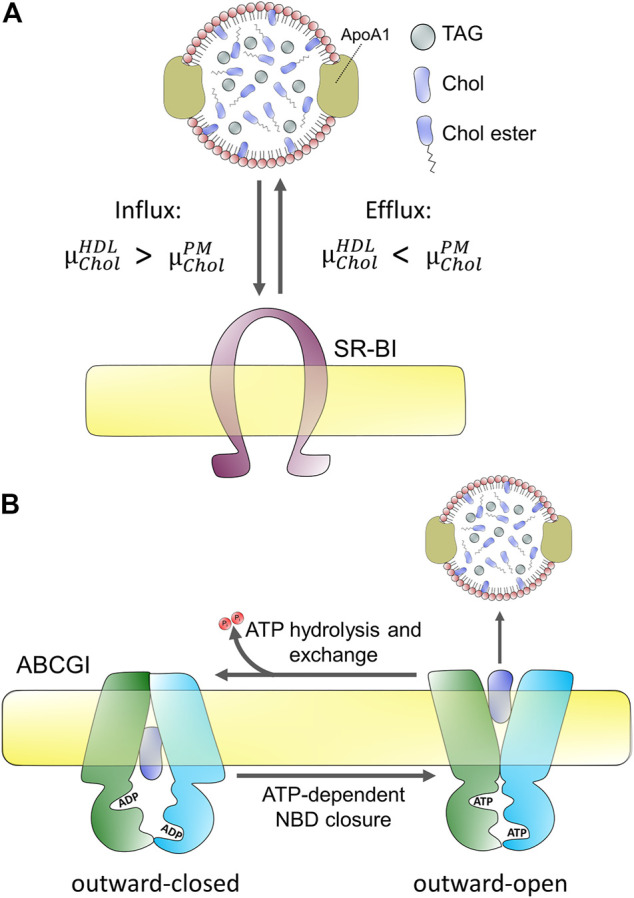
Passive and active protein-mediated cholesterol efflux from mammalian cells. **(A)** passive efflux does not require hydrolysis of ATP but depends on the difference of chemical potentials of cholesterol in the acceptor, here HDL $\left(\mu_{Chol}^{HDL}\right)$ and PM $\left(\mu_{Chol}^{PM}\right)$ . Cholesterol efflux requires, that $\mu_{Chol}^{HDL}$ < $\mu_{Chol}^{PM}$ , and is shown here for scavenger receptor BI (SR-BI). **(B)** Active efflux, illustrated here for the ABC transporter ABCG1, depends on the binding of two ATP molecules, which stabilize an outward-open conformation for efflux of cholesterol and phospholipids to e.g., HDL. ATP-hydrolysis causes the transition of the protein two an outward-closed conformation, allowing for entrance of new substrate on the cytosolic side of the PM. The full cycle is more complex and involves at least four steps; see (Skarda et al., 2021).

Early observations made more than 50 years ago showed that cholesterol can be partly removed from mammalian cells by incubation with serum and particularly the high-density lipoprotein (HDL) fraction of serum (Bailey, 1965; Rothblat et al., 1999). Later studies showed that the half-time of cholesterol efflux is cell-type dependent, being about 4 h in rat hepatoma cells but more than 1 day in human fibroblasts (reviewed in Rothblat et al. (1999)). Cells can efflux cholesterol to the HDL apoprotein apoA1, to lipid vesicles, and to serum albumin (Rothblat et al., 1999; Sankaranarayanan et al., 2013). Additionally, cyclodextrins have been shown to be efficient efflux acceptors, often employed as research tools to study kinetic pools of cellular cholesterol efflux (Kilsdonk E. P. C. et al., 1995; Yancey et al., 1996; Haynes et al., 2000). While the kinetics of cholesterol efflux differs between sterol acceptors, the overall rank order of efflux between cell types does not, and only the abundance of phospholipids in acceptor particles seem to dictate the net efflux capacity (Rothblat et al., 1999). To a large extent, the formation of nascent HDL takes place at the cell surface. However, HDL can also receive cholesterol upon endocytosis of the lipoprotein, its passage through early endosomes, and recycling back to the cell surface, as shown for mature HDL and apoprotein E (Heeren et al., 2003; Heeren et al., 2004). This so-called retroendocytosis can also deliver cholesterol from HDL to cells as shown in fibroblasts and hepatocytes by biochemical methods and quantitative imaging, respectively (Webb et al., 2004; Wüstner et al., 2004; Wüstner, 2005; Sun et al., 2006; Röhrl et al., 2012). It appears that net cholesterol efflux is only possible if the chemical potential of cholesterol in the acceptor particles, for example HDL, $\mu_{Chol}^{HDL}$ , is lower than in the donor membrane, which could be the PM, $\mu_{Chol}^{PM}$ , (Yancey et al., 1996; Rothblat et al., 1999). In the opposite case, cholesterol will be delivered to cells, and if the chemical potentials are equal, i.e., $\mu_{Chol}^{HDL}$ = $\mu_{Chol}^{PM}$ , cholesterol will passively exchange between donor and acceptor without net efflux from or net influx into cells (Figure 2).

### 4.2 Cells Regulate Cholesterol Efflux by Setting its Chemical Potential in Membranes and Lipoproteins

How can cells regulate the efflux of cholesterol based on this physicochemical principle? There are primarily two mechanisms mammalian cells use to ensure efflux of cholesterol by lowering its chemical potential in the acceptor particles; 1) the fraction of phospholipids in acceptor particles is increased, providing interaction partners for cholesterol, thereby lowering its chemical potential, and 2) by preventing cholesterol from being recaptured by cells via its esterification in the acceptor particle (Fielding, 2009). Both processes are widely implemented and—in fact—tightly regulated, as will be discussed in more detail below. First, the efflux efficiency of sera is strongly correlated with HDL phospholipid levels and many efflux transporters, such as ABC transporters either co-transport phospholipids with cholesterol or even have certain phospholipid species as primary substrate (Rothblat et al., 1999; Coleman et al., 2013). Second, Lecithin:cholesterol acyltransferase (LCAT) catalyzes the transfer of an acyl chain from PC, the main phospholipid in cell membranes and lipoproteins, to the released cholesterol, thereby creating CEs and lysolecithin in the acceptor particle (Fielding and Fielding, 1971; Fielding, 1985). Conversion of cholesterol into CEs in HDL lowers its chemical potential, $\mu_{Chol}^{HDL}$ , ensuring that the condition for efflux of cholesterol from cells along its chemical potential gradient (i.e., $\mu_{Chol}^{HDL}$ < $\mu_{Chol}^{PM}$ ) is maintained (Figure 2). Electron microscopy (EM) combined with cross-linking and deuterium exchange mass spectrometry has been used to show that LCAT directly binds to nascent, discoidal HDL particles via interaction with the protein belt formed by helix 5 and 6 of apoA1 (Nakamura et al., 2004; Manthei et al., 2020). Importantly, the activity of the enzyme is highest for lipid-poor apoA1 containing 2–3 PC and cholesterol molecules compared to mature HDL and ceases, as HDL particles mature (Nakamura et al., 2004; Fielding, 2009). This is likely, because a lid at the LCAT surface, which is necessary to allow for substrate entrance and activation by lipid-free apoA1, is prevented from opening in mature HDL particles but not in nascent HDL (Manthei et al., 2017; Manthei et al., 2020). Thus, biochemical feedback ensures that lipoprotein-associated cholesterol esterification ceases, when HDL has matured, and efflux stops (i.e. $\mu_{Chol}^{HDL}$ ≈ $\mu_{Chol}^{PM}$ ). Subsequently, the sterol-loaded HDL particle can return to the liver for transfer of CEs into hepatocytes followed by cholesterol secretion into bile or incorporation of cholesterol into very low density lipoproteins (VLDL) (Robins and Fasulo, 1997; Wanon et al., 1998; Robins and Fasulo, 1999). A third possible mechanism to maintain a chemical gradient of non-esterified cholesterol between cells and efflux acceptors, such as HDL is to remove some of the generated CEs in exchange for PC with other lipoproteins. This process is catalyzed by cholesteryl ester transfer protein (CETP), which exchanges CEs for PC molecules in a 1:1 fashion between HDL and apoprotein B containing lipoproteins, such as VLDL and LDL (Tall, 1993; Barter et al., 2003). Unfortunately, enrichment of these lipoproteins with CEs has a proatherogenic effect, which made inhibitors of CETP an attractive target for drug development (Ferri et al., 2018). However, despite several promising drug candidates as CETP inhibitors, none have been approved (Sheridan, 2016). Anacetrapib, developed by Merck, showed particularly promising results but was in the end not submitted for regulatory approval, as the drug had a very long half-life (Nissen, 2017). Other drugs, such as torcetrapib developed by Pfizer and Dalcetrapib developed by Roche, also raised plasma HDL levels but did not show any benefit in preventing cardiovascular disease (Sheridan, 2016). Based on these outcomes, it is unlikely that developing CETP inhibitors is a useful strategy for treatment of arteriosclerosis and cardiovascular disease in the near future (Swain, 2017). In some cases, HDL might also directly receive cholesterol from other lipoproteins, for example from aggregated LDL during contact with activated macrophages in the intima of the vessel wall (Singh et al., 2019).

## 5 Structure, Membrane Dynamics, and Molecular Function of Cholesterol Efflux Transporters

### 5.1 Transporters for Active Versus Passive Efflux of Cholesterol From Cells

Since cholesterol is very hydrophobic its water solubility is very low (i.e., below 30 nM), and there is a high free energy cost of moving it between membranes through the water phase (Rothblat et al., 1999; Wüstner and Solanko, 2015). Consequently, cholesterol exchange between membranes is very slow in the absence of proteins, and mammalian cells use a variety of transporters to facilitate cholesterol efflux to lipoproteins. Energy-dependent efflux of cholesterol from cells is mediated by ABC transporters, primarily ABCA1, ABCG1, and ABCG4 (Tall et al., 2002; Phillips, 2014). Passive cholesterol exchange between cells and mature HDL is facilitated by scavenger receptors, especially scavenger receptor BI (SR-BI; Figure 2), but results concerning the quantitative contribution of each of these transporters are not without contradiction (Wang et al., 2001; Thuahnai et al., 2003; Peng et al., 2004; Wang et al., 2004; Yancey et al., 2004a; Yancey et al., 2004b; Vedhachalam et al., 2007; Wang et al., 2007b; Pagler et al., 2011). Cell-type specific differences reflected in different tissue expression of these transporters contribute to disparate findings concerning their precise role in cholesterol efflux. In addition, they might have a varying affinity to nascent versus mature HDL. For example, while ABCG1/4 and SR-BI preferentially transfer cholesterol to mature HDL, ABCA1 plays a major role in the initial lipidation of ApoA1 to form nascent HDL particles (Liu et al., 2003; Thuahnai et al., 2003; Wang et al., 2004; Vaughan and Oram, 2006; Adorni et al., 2007). Also, ATP-dependent efflux by ABC transporters, such as ABCA1 and exchangers, like SR-BI seems to be inversely regulated (Chen et al., 2000). Based on these and other findings, a model has been proposed according to which the specific interaction between ApoA1 and ABCA1 creates nascent HDL particles which become transformed into mature HDL, increasingly receiving cholesterol from efflux by ABCG1 and ABCG4 (Vaughan and Oram, 2006; Wang et al., 2008). SR-BI is a bi-directional transporter with regulated and cell-type dependent endocytosis and recycling (Silver et al., 2001; Eckhardt et al., 2004; Webb et al., 2004; Wüstner, 2005; Shetty et al., 2006; Sun et al., 2006; Marques et al., 2019). Most cholesterol transfer between HDL and SR-BI seems to take place at the cell surface, but some cholesterol exchange can also happen in endosomes (Wüstner et al., 2004; Wüstner, 2005; Sun et al., 2006; Marques et al., 2019). The direction of net cholesterol flux between subcellular membranes is also dictated by the sterol gradient, such that cholesterol moves from membranes where its chemical potential is high to membranes, where its chemical potential is low (see above and Figure 2). Therefore, efflux of cholesterol to an acceptor lowers the chemical potential of cholesterol in the donating membrane (e.g., the PM), which, in turn, stimulates replenishment of cholesterol from intracellular sites. Vice versa, selective uptake of cholesterol from HDL will increase the chemical potential of cholesterol in the PM, thereby stimulating non-vesicular sterol transfer to intracellular sites. SR-BI also mediates selective uptake of cholesterol and CEs from HDL into hepatocytes, which plays an important role in cholesterol clearance into bile during reverse cholesterol transport (Kozarsky et al., 1997; Robins and Fasulo, 1997; Robins and Fasulo, 1999). Similarly, SR-BI mediates selective uptake of CEs into steroid hormone-producing cells. Based on the crystal structure of LIMP-2, which is a related member of the CD36 superfamily of scavenger receptors, a large hydrophobic cavity acting as a tunnel for transfer of CEs has been proposed for SR-BI (Neculai et al., 2013). Bidirectional lipid transfer between membranes and HDL or other lipoproteins can also be induced as a passive process upon contact, as shown by combined fluorescence correlation spectroscopy and high-speed atomic force microscopy (AFM) (Plochberger et al., 2017). Thus, the function of SR-BI and other scavenger receptors might be primarily to bring lipoprotein and PM into close enough contact, such that hydrophobic lipid transfer can take place. Alternatively, the hydrophobic channel formed by SR-BI is needed for sterol exchange. Future studies are needed to clarify this issue.

### 5.2 ATP-Binding Cassette Transporter A1 is a Prototype Efflux Transporter for Cholesterol

Mutations in ABCA1 have been associated with Tangier disease, a rare genetic disorder characterized by very low levels of HDL and apoA1 and high accumulation of CEs, primarily in blood-derived macrophages but also in tissue macrophages in various organs, such as tonsils, liver, spleen, and lymph nodes (Tall and Wang, 2000; Quazi and Molday, 2011). Additionally, patients have lipid deposits in other cell types, such as fibroblasts, Schwann cells, or smooth muscle cells (Oram, 2000). Symptoms of Tangier disease include coronary artery disease, neuropathies, splenomegaly, and hepatomegaly (i.e., enlargement of spleen and liver) (Oram, 2000). A recently reported cryo-electron microscopy structure of ABCA1 with 4.1 Å resolution revealed the symmetric nature of the protein, in which the two transmembrane domains with the connected nucleotide-binding domains and the two large extracellular domains form a hydrophobic tunnel for lipid translocation (Qian et al., 2017). A lateral access model for lipid substrates has been proprosed and Tangier disease mutations could be mapped to the structure of the protein (Qian et al., 2017). Interestingly, monomeric ABCA1 has significant sequence identity and similarity with ABCG5/8, a heterodimer of ABC transporters, which is responsible for the excretion of cholesterol from the apical canalicular membrane of hepatocytes into bile (Yu et al., 2002; Qian et al., 2017). Both ABC structures can be superimposed revealing close structural similarity despite the fact, that they belong to different ABC transporter subfamilies (Qian et al., 2017). ABCA1 might act in concert with other ABC transporters, such as ABCG transporters, but also with ABCA8 and ABCA12, as shown in macrophages during reverse cholesterol transport (Fu et al., 2013; Trigueros-Motos et al., 2017). More information about the structure, regulatory sequences, such as phosphorylation and ubiquitination sites, as well as substrate specificity of ABCA1 and other ABC transporters involved in cholesterol transport can be found in comprehensive recent reviews (Phillips, 2018; Kerr et al., 2021).

### 5.3 Quantitative Imaging of Membrane Transporters for Cholesterol Efflux

Live-cell imaging and correlative microscopy have contributed greatly to our current understanding of proteins involved in cholesterol export from cells. For example, by combining EM with light microscopy using diaminobenzidine induced photooxidation, Stangl and co-workers studied subcellular trafficking of SR-BI and its lipid substrates with high resolution (Röhrl et al., 2012). Recent single-molecule tracking and Förster resonance energy transfer (FRET) imaging experiments in living cells revealed that SR-BI is primarily organized in dimers and fails to enter clathrin-coated pits for endocytosis (Sahoo et al., 2007; Marques et al., 2019). This underlines its importance in exchange of cholesterol and CEs at the cell surface. Green fluorescent protein-tagged ABCA1 (GFP-ABCA1) traffics very dynamically between PM, endo-lysosomes, and the Golgi apparatus in cells (Neufeld et al., 2001; Neufeld et al., 2004). GFP-ABCA1 diffuses apparently freely in the PM, but its lateral dynamics depends on its ATPase activity and binding to apoA1, as shown by spot variation fluorescence correlation spectroscopy (Raducka-Jaszul et al., 2021). Similarly, the ATPase activity of ABCA1 affects the diffusion of other membrane proteins, such as the transferrin receptor and fluorescent lipid analogues, as shown in fluorescence recovery after photobleaching experiments (Zarubica et al., 2009). GFP-ABCA1 resides in liquid-disordered domains in formaldehyde-induced giant plasma membrane vesicles (GPMVs), suggesting that it prefers a fluid lipid environment (Zarubica et al., 2009). Single-molecule tracking found GFP-ABCA1 in largely immobile spots at the PM, and its confinement depended on its ATPase activity, somehow contradicting the findings made by fluorescence correlation spectroscopy (Nagata et al., 2013; Raducka-Jaszul et al., 2021). Photobleaching of GFP-ABCA1 occurred primarily in two discrete steps, indicating that the protein forms dimers in the PM, supporting an earlier study based on FRET imaging and native PAGE analysis (Trompier et al., 2006; Nagata et al., 2013). Both studies found that apoA1 stabilizes dimers or oligomers, suggesting that ABCA1 is not able to transfer lipids to apoproteins in its monomeric state. These and similar studies show the power of sensitive fluorescence imaging to reveal the molecular function and dynamics of ABC transporters in cholesterol efflux from cells (Wong et al., 2016). But it remains an important challenge to validate results of membrane receptor dynamics with different imaging approaches.

## 6 Mechanisms of Lipid Efflux to apoA1—Which Structures are Formed at the Cell Surface?

### 6.1 ATP-Binding Cassette Transporter A1 Transports a Variety of Phospholipids Across Lipid Membranes

Several hypotheses have been put forward to explain the molecular mechanisms underlying ABCA1’s function: 1) ABCA1 acts as a receptor for apoA1 on the cell surface thereby catalyzing the direct transfer of lipids onto apoA1 (Wang et al., 2001; Fitzgerald et al., 2004). 2) ABCA1, which follows a complex intracellular trafficking scheme (Neufeld et al., 2002; Zha et al., 2003; Neufeld et al., 2004), mediates lipidation of apoA1 during its passage through the cell, likely by a retroendocytic pathway (Denis et al., 2008). 3) ABCA1 acts as a pump for lipids, like the aminophospholipid PS, or other hydrophobic substances at the cell surface, which is supported by its intrinsic ATPase activity and substrate specificity (Wang et al., 2001; Tall et al., 2002; Alder-Baerens et al., 2005; Linsel-Nitschke and Tall, 2005; Quazi and Molday, 2013), 4) ABCA1 acts as cholesterol floppase, i.e. actively moving cholesterol from the inner to the outer PM leaflet for pick up by apoA1 (Ogasawara et al., 2019; Okamoto et al., 2020). In fact, ABCA1 translocates analogues of PC, SM, and PS across membranes in a reconstituted system and PE and PS to the outer leaflet of mammalian cells, and it seems to bind and transport even phosphatidylinositol-bisphosphate (Alder-Baerens et al., 2005; Quazi and Molday, 2013; Gulshan et al., 2016). Whether ABCA1 directly binds cholesterol, a precondition for its translocation by this ABC transporter, is debated (Reboul et al., 2013; Dergunov et al., 2019).

### 6.2 ATP-Binding Cassette Transporter A1 Controls the Transbilayer Distribution of Lipids in the Plasma Membrane

Phospholipid asymmetry in the PM has a regulatory function in cholesterol efflux, and there is a large body of evidence that ABCA1 affects the transverse distribution of phospholipids and cholesterol in the PM, thereby increasing the propensity of these lipids to efflux from cells. Translocation of negatively charged phospholipids to the outer PM leaflet by ABCA1 causes altered surface membrane potential and reduced rate of endocytosis (Zha et al., 2001; Alder-Baerens et al., 2005; Zarubica et al., 2009). Exposing more PS to the outer leaflet by other mechanisms, for example by inhibiting the synthesis of sphingolipids with myriocin, causes increased ABCA1-independent cholesterol efflux in RAW macrophages (Gulshan et al., 2013). The same treatment increased the ergosterol content in the outer PM leaflet of yeast cells and long-chain sphingolipids in the outer leaflet regulate cholesterol asymmetry in the PM of mammalian cells (Courtney K. C. et al., 2018; Solanko et al., 2018). Thus, it is likely that altering the transbilayer distribution of PS and abundance of sphingolipids can indirectly cause an increased availability of cholesterol for efflux from the outer PM leaflet. Interestingly, the expression of ABCA1 shifts the steady state distribution of not only PS but also of fluorescent cholesterol analogues towards the outer leaflet, from which apoA1 can directly access the sterols for efflux (Alder-Baerens et al., 2005; Pagler et al., 2011; Gulshan et al., 2013). Since the transbilayer dynamics of cholesterol in model membranes and likely the PM of living cells is very high, with flip-flop time constants in the msec-sec range, any actively generated cholesterol gradient across the PM bilayer would equilibrate rapidly (John et al., 2002; Steck et al., 2002; Bennett et al., 2009). Measured hydrolytic activity of ABCA1 and other ABC transporters for ATP is too slow to maintain a transbilayer cholesterol asymmetry by directly pumping sterol to the outer leaflet in the presence of such high rates for passive sterol flip-flop (Quazi and Molday, 2013; Skarda et al., 2021). This is in contrast to the much slower passive flip-flop of phospholipids across membranes (Coleman et al., 2013). Thus, instead of directly transporting cholesterol, it is more likely that active translocation of PS by ABCA1 indirectly increases cholesterol availability in the outer half of the bilayer for efflux to acceptor proteins. Supporting that notion are observations in yeast and mammalian cells, which found that loss of PS asymmetry or abundance in the inner leaflet increases cholesterol availability in the outer leaflet of the PM (Maekawa and Fairn, 2015; Solanko et al., 2018). Together, these results suggest that ABCA1 alters the lipid composition and transbilayer orientation of lipids in the PM, which can facilitate phospholipid and cholesterol efflux from cells to apoA1.

### 6.3 Does ATP-Binding Cassette Transporter A1 Facilitate Protrusion of Cholesterol From the Bilayer or Bending of the Plasma Membrane?

Binding experiments with fluorescence-labeled PFO, which detects membrane cholesterol above a characteristic threshold concentration, found strong binding in cells expressing ABCA1 (Ogasawara et al., 2019; Okamoto et al., 2020). The cholesterol pool detectable by PFO domain D4 and similar reporters, such as the cholesterol-binding domain 4 of anthrolysin O (ALO-D4), has often been associated with “accessible” or “active” cholesterol (see Section 3, above) (Courtney K. C. et al., 2018). The increased chemical potential of “active cholesterol” implies, that it might have an elevated propensity to protrude from the membrane, but whether this sterol pool is entirely available for efflux from cells remains to be shown. It also remains an open question whether the ATPase activity of ABCA1 is only used to translocate PS and other phospholipids to the outer PM leaflet, or also to decrease the energy barrier for partial cholesterol protrusion from the bilayer, which could facilitate sterol transfer to an acceptor particle, such as apoA1 (Small, 2003; Van Meer et al., 2006; Phillips, 2014; Plummer et al., 2021). Zha and co-workers have shown that expression of ABCA1 without any addition of apoA1 changes the organization of proteins and lipids in the PM and causes the release of apoA1-free microparticles with a size of >20 nm (Landry et al., 2006; Nandi et al., 2009). RAW macrophages express ABCA1 only when preincubated in the presence of cAMP to activate protein kinase A, and expression of functional ABCA1 in the absence of apoA1 triggers cellular excretion of apoA1-free microparticles (Liu et al., 2003; Nandi et al., 2009). Vice versa, deletion of ABCA1 and ABCG1 resulted in increased cholesterol content of the PM and impaired migration of macrophages due to increased Rac signaling (Pagler et al., 2011). Morphological analysis of EM images of baby hamster kidney (BHK) cells with inducible expression of ABCA1 revealed disc-like HDL particles with a diameter < 20 nm and additionally larger vesicular structures secreted from cells with a diameter of 50–150 nm and up to 500 nm (Hafiane and Genest, 2017). Such microparticles were also secreted by THP1 macrophages in an ABCA1 and ApoA1 dependent manner (Hafiane and Genest, 2017). Could such particles be vesicles which form at the PM and if so, how do they form? When apoA1 inserts into the lipid bilayer, formation of an “activated lipid domain” has been proposed, which can lead to outward-directed vesiculation of the PM (Vedhachalam et al., 2007; Phillips, 2014). Such a mechanism could be supported by ABCA1-mediated translocation of phospholipids to the outer PM leaflet, creating increased lateral pressure in this monolayer thereby facilitating membrane bending (Coleman et al., 2013; Phillips, 2014). Active lipid flipping has been proposed as a mechanism for the regulation of endocytosis by aminophopholipid translocases, which catalyze ATP-dependent flipping of PS and PE to the inner PM leaflet (Pomorski et al., 2003; Hirama et al., 2017). Since ABC transporters, such as ABCA1 transport phospholipids in the opposite direction, they can create an excess area in the outer PM monolayer, and this asymmetric membrane stress can be relieved by bending the bilayer outwards, eventually leading to the formation of exovesicles and/or nanodiscs (Coleman et al., 2013). Such a monolayer pleating effect of phospholipid flopping to the outer leaflet is supported by molecular dynamics simulations (Segrest et al., 2015). This study suggests that ABCA1 forms an extracellular reservoir containing an isolated squeezed monolayer, which triggers the release of membrane particles from the PM. In the presence of apoA1, such particles are primarily membrane discs, with a majority of lipids originating from the outer PM leaflet, while in the absence of ApoA1, the disks transform into unilamellar vesicles (Segrest et al., 2015). In this model, tight packing of membrane helices of ABCA1 in dimers could control access of cholesterol to prevent it from counterbalancing the outward bending of the bilayer. The latter is needed since fast passive flipping of cholesterol to the inner leaflet was found to relax bending energies of membranes, both in experiments and simulations (Bruckner et al., 2009; Choubey et al., 2013; Segrest et al., 2015). Whether ABCA1 controls access of cholesterol to the forming membrane protrusions, as suggested in simulations (Segrest et al., 2015), or eventually reduces its flipping to the inner leaflet during membrane bending by a cholesterol floppase activity remains to be determined (Okamoto et al., 2020). In our view, the latter is unlikely due to the high passive flip-flop rates of cholesterol and the comparably slow ATPase activity of ABC transporters (see above).

### 6.4 Biliary Lipid Secretion as a Model for Exo-vesiculation of the Plasma Membrane by ATP-Binding Cassette Transporters

Interestingly, a very similar vesiculation mechanism to that described above has been proposed to mediate biliary secretion of cholesterol and PC. This process depends on the ABCG5/8 heterodimer and the PC-translocating ABC transporter ABCB4, also named multidrug resistance protein 2 (MDR2), which are both expressed in the canalicular membrane of hepatocytes (Yu et al., 2002). ABCG5/8 mediate the secretion of cholesterol and plant sterols, such as sitosterol, into bile and intestine, and mutations in these transporters lead to sitosterolemia, an inherited accumulation of plant sterols in various tissues (Plummer et al., 2021). Loss of functional ABCG5/8 or ABCB4 leads to defective lipid secretion into the bile, which is the major pathway for cholesterol clearance from the human body (Small, 2003). In mice, about 55% of cholesterol is excreted as fecal neutral sterols, with the remainder being secreted as bile acids (Dietschy and Turley, 2002). However, these values can change dramatically in animals with defective sterol transporters. EM of rapidly frozen liver sections, has provided evidence that the initial event of biliary secretion of PC and cholesterol takes place as small vesicles, shed directly from the canalicular membrane (Crawford et al., 1995). This process is facilitated by the aforementioned ABC transporters and by bile acids, cholesterol-derived detergents, which contribute to lipid solubilization in the bile fluid (Crawford, 1996; Crawford et al., 1997; Small, 2003). Bile acids intercalate into the outer leaflet of the PM, from which they can solubilize phospholipids, either as mixed micelles or as microvesicles, and independent of phospholipid head group composition (Kuipers et al., 1997; Wüstner et al., 1998; Wüstner et al., 2000). Thus, a preferred vesiculation of the outer leaflet of the canalicular membrane was proposed to result in specific enrichment of PC and cholesterol in the bile fluid (Crawford et al., 1995; Small, 2003). Monomeric bile acids can also directly stimulate the ATPase activity of ABCB4/MDR2 (Kroll et al., 2021). Active translocation of PC to the outer leaflet by ABCA4 as well as of aminophospholipids to the inner leaflet of the canalicular membrane by the P-type ATPase ATP8B are required to prevent membrane damage and to ensure specific enrichment of PC, bile acids, and cholesterol in the bile (Tannert et al., 2003; Cai et al., 2009; Groen et al., 2011). This conclusion is supported by clinical manifestations of cholestasis and liver damage when either ABC4 or ATP8B are mutated and dysfunctional, as observed in progressive familial intrahepatic cholestasis (Cai et al., 2009; Groen et al., 2011). Another example for cooperation between ABC transporters in lipid secretion is that of ABC4 and ABCG5/8, which are both required for release of cholesterol into the bile (Langheim et al., 2005). Interestingly, while apoA1 secreted into bile can assist in solubilizing cholesterol and preventing gallstone formation (Secknus et al., 1999), the ABCA1/apoA1 system is not directly involved in cholesterol secretion from hepatocytes into bile. In fact, ABCA1 resides in the basolateral but not in the canalicular membrane in cultured polarized hepatocytes (Neufeld et al., 2002). ABCA1 is also enriched in a subapical endocytic compartment in such hepatocytes, an organelle which is known to be rich in free cholesterol and accessible to HDL from the basolateral cell surface during endocytic recycling (Silver et al., 2001; Neufeld et al., 2002; Wüstner et al., 2002; Wüstner et al., 2004). Consequently, ABCA1 is likely involved in regulating intracellular hepatic cholesterol and plasma HDL levels (Neufeld et al., 2002).

## 7 Origin, Structure, and Function of Cholesterol-Containing Extracellular Vesicles and Particles

### 7.1 The Diversity of Extracellular Vesicles and the Challenge of Their Classification

**FIGURE 3:**
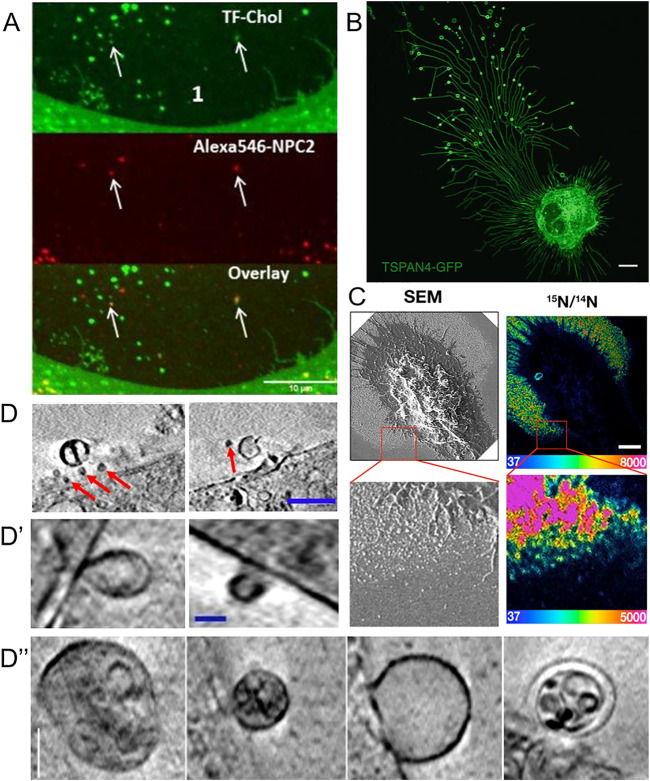
Visualization of extracellular vesicles and particles during cholesterol efflux. **(A)** Spinning disk microscopy image of extracellular vesicles from a NPC2-deficient fibroblasts treated with NPC2 protein, tagged with a red Alexa546 fluorophore (red), and labeled additionally with TopFluor-cholesterol (green) (Juhl et al., 2021). **(B)** Confocal microscopy image of migrasomes from L929 cell transfected with TSPAN4-GFP (Chen et al., 2018). Scalebar 10 µm. **(C)** SEM of nanoSIMS of macrophage after incubation with [^15^N]ALO-D4. Scalebar 2 µm (He et al., 2018). **(D,D′,D′′)** examples of extracellular vesicles from NPC2 deficient and healthy fibroblasts imaged with cryo-SXT. Scalebar 0.5 µm for **(D)** and 0.15 µm in **(D′,D′′)** (Juhl et al., 2021). Red arrows point to microvesicles.

Extracellular vesicles formed independently of the HDL/ABC transporter system have started to be recognized as cholesterol efflux routes, eventually complementing the classical efflux pathways (Pfrieger and Vitale, 2018). Based on their size and origins, extracellular vesicles have roughly been divided into exosomes and microvesicles. Exosomes originate from ILVs inside multivesicular bodies which can fuse with the PM during lysosomal exocytosis (Gruenberg, 2020). They are typically in the size range of 30–150 nm, and their secretion contributes to the efflux of excess cholesterol in lysosomal storage disorders (see below, 8.) (Strauss et al., 2010; Vacca et al., 2019; Ilnytska et al., 2021a). Microvesicles, also called ectosomes, are generated by outward budding of the PM and tend to be more heterogeneous in their morphology and content compared to exosomes (Cocucci et al., 2009). Microvesicles tend to be larger, normally around 100–1,000 nm in size, but have been reported to be up to 8–10 µm in size (Falchi et al., 2013). However, the classification of extracellular vesicles should be taken with some caution, as no standard approaches for their characterization have yet been defined (Cocucci et al., 2009; Van Niel et al., 2018). Several methods and approaches are being used to examine extracellular vesicles. One way is to first isolate the vesicles, and subsequently analyze them. The isolation of cell-derived vesicles can be done by several methods including centrifugation, filtration, and aqueous two-phase systems, and the analysis can be carried out with techniques such as immunoblotting, dynamic light scattering, and microscopy (Kim et al., 2015; Shin et al., 2015; Pollet et al., 2018; Hartjes et al., 2019; Kırbaş et al., 2019; Mondal et al., 2019). Another approach is to study the biology of extracellular vesicles directly by microscopy as discussed below and recently reviewed by Verweij et al. (2021). He et al. (2018) used a combination of scanning electron microscopy (SEM) and nanoscale secondary ion mass spectrometry (nanoSIMS) to study PM-derived particles secreted from macrophages upon loading with acetylated LDL (He et al., 2018). Their findings confirm and extend earlier observations made by EM and particle size analysis (Hafiane and Genest, 2017). SEM allowed He et al., 2018) to visualize the formation and release of the particles, whereas nanoSIMS enabled them to obtain high-resolution images of the particle’s cholesterol content (Figure 3). With this approach, the authors found that the cholesterol content of such released particles varies depending on the cholesterol loading condition (He et al., 2018). By growing the macrophages in the presence of fetal bovine serum and by activating cholesterol efflux via stimulation of LXRs and RXR complexes with agonists, He et al. found that the particles became enriched with cholesterol accessible to ALO-D4, and that this cholesterol pool could be transferred to HDL (He et al., 2018).

### 7.2 Does Cholesterol Aggregate During its Efflux From Cells?

Using an antibody named mAb 58B1, which is supposed to bind specifically to two-dimensional arrays of 10–20 cholesterol molecules, Jin et al. (2018) reported that macrophages remove excess cholesterol by shedding of cholesterol microdomains (Figure 3A) (Jin et al., 2018). This “type” of shedded cholesterol, was different from the one described by He et al. (2018), as particles tended to be larger, up to several hundred nm, and irregularly shaped rather than spherical, like vesicles. The authors of this study found that the cholesterol-binding polyene filipin does not label the cholesterol microdomains detected with mAb 58B1, and they speculated that filipin cannot intercalate into the supposed two-dimensional arrays of cholesterol in the PM of cholesterol-loaded macrophages (Jin et al., 2018). An alternative explanation would be that the primary IgM antibody used in this study cross-links membrane-dispersed cholesterol due to its multivalency, which could be further enhanced by the use of secondary antibodies, employed for detection. Thus, lateral cholesterol clustering could be induced by the antibody treatment, and there is no evidence for naturally occurring cholesterol microdomains. Ample evidence obtained by fluorescence imaging of fluorescent cholesterol analogues shows that sterols move rapidly and freely in the PM of living cells and do not form clusters detectable by light microscopy and super-resolution microscopy (Wüstner et al., 2016; Pinkwart et al., 2019). Even in cholesterol-loaded cells, such as macrophage foam cells, in which ACAT was inhibited, fluorescent cholesterol analogues did not cluster in the PM (Wüstner, 2008). On the other hand, Kruth, Addadi and co-workers found by super-resolution fluorescence microscopy of antibody-treated cells and by X-ray microscopy that microcrystals can form in macrophages under prolonged excess cholesterol loading conditions in the absence of sterol acceptors (Addadi et al., 2003; Varsano et al., 2016). Formation of cholesterol crystals in macrophages after extended incubation with excess cholesterol or atherogenic lipoproteins has been shown previously by EM, and this might be related to the occurrence of cholesterol crystals in the intima of the vessel wall in patients suffering from myocardial infarction (Tangirala et al., 1994; Kellner-Weibel et al., 1999; Janoudi et al., 2016). It is possible, that excess cholesterol precipitates from the membrane to form cell-adhered crystals, if cholesterol efflux mechanisms fail. Release of cholesterol in microparticles, as reported by Kruth and co-workers depended on ABCA1, as the authors found no, or very few, microparticles released from macrophages treated with the ABCA1 inhibitor, probucol (Jin et al., 2018). Future studies are needed to clarify the nature of different microparticles released from macrophages during cholesterol efflux.

### 7.3 Is Shedding of Cholesterol-Rich Microvesicles Coupled to Cell Migration?

Using a combination of proteomics, tracer experiments, super-resolution microscopy, SEM, live cells imaging, and nanoSIMS, Young and co-workers showed that the membrane-derived particles, they described earlier, were released from the macrophages during cell migration (He et al., 2018; Hu et al., 2019). The authors also showed that the released particles were enriched with ALO-D4 accessible cholesterol, but not sphingolipid-sequestered cholesterol (Figure 3) (Hu et al., 2019). Interestingly, Das et al. showed that the cholesterol content of the plasma membrane can be divided into three distinct pools: a pool accessible to bacterial toxins, a pool sequestered by sphingomyelin, and an essential pool (Das et al., 2014). The accessible pool was found to be connected with the cholesterol levels in the ER and thus is involved in cellular cholesterol homeostasis (Das et al., 2014; Infante and Radhakrishnan, 2017). Hu et al. suggested that the sphingolipid-sequestered cholesterol, located in the outer leaflet of the PM, is more likely to remain in the membrane during movement as it is indirectly associated with the actin cytoskeleton (Raghupathy et al., 2015; Hu et al., 2019). The toxin-accessible cholesterol pool of the PM could be more prone to be released from cells during migration (Infante and Radhakrishnan, 2017; Hu et al., 2019). Thus, it is likely that the movement of cells, and thereby the release of cholesterol-rich particles contributes to cellular cholesterol efflux. Supporting that notion, the release of membrane fragments in the form of vesicles and nanotubes during cell migration has been known for decades (Huttenlocher et al., 1995; Fuhr et al., 1998). Such cell traces have been visualized by a variety of imaging techniques, including confocal, interference reflection, and total internal reflection microscopy as well as by AFM and EM (Fuhr et al., 1998). Support for cholesterol efflux from cells in surface-shed vesicles during migration comes from other recent imaging studies; using an elegant mixture of live-cell imaging, EM, and mass spectrometry, Ma et al. described a migration-dependent mechanism of vesicle release from cells, named migracytosis (Ma et al., 2015). During migration, the cell will leave retraction fibers behind on which vesicles up to 3 µm in size can grow, either at the fiber tips or at fiber intersections (Figure 3B). These vesicles, named migrasomes, will eventually detach from the fibers and be released. The migrasomes contain a varying number of smaller vesicles and during their growth, they are actively receiving content from the main cell body (Huang et al., 2019). They contain increased amounts of the protein tetraspanin 4 (TSPAN4) and more cholesterol than the retraction fibers, and both, cholesterol and TSPAN4, were necessary and sufficient for migrasome formation (Huang et al., 2019). The primary function of migrasomes has been suggested to be in cell-cell communication (Ma et al., 2015). But due to their relatively high content of cholesterol, their detachment from the cell could be another indirect way for the cell to release cholesterol. Excess cholesterol can also be transferred to co-cultured smooth muscle cells in the absence of HDL or serum (He et al., 2020). Using nanoSIMS, Young and co-workers showed that this pathway is substantially contributing to cholesterol efflux from macrophages and does not depend on ABCA1 (He et al., 2020). Whether it relies on shedding of microvesicles from the donor cells, remains to be determined in future studies. Another recent study reported shedding of large GPMVs containing the cholesterol markers TopFluor-cholesterol or filipin, in human fibroblasts upon induction of membrane vesiculation using dithiotreitol (Sedgwick et al., 2018). Formation of such GPMVs was enhanced upon cyclodextrin treatment to remove cholesterol and upon microtubule stabilization and required actin polymerization (Sedgwick et al., 2018). However, GPMVs are often employed as a model system for the PM, and their chemical-induced formation does not resemble natural vesiculation processes taking place in intact and healthy cells (Sezgin et al., 2012). Table 1 gives an overview of described extracellular cholesterol-rich particles, their size, cellular origin, and method of detection with focus on various imaging studies.

**TABLE 1:** Overview of reported extracellular non-lipoprotein particles rich in cholesterol.

Particle type	Size	Method	Cell type	Reference
Membrane-derived particles	30–250 nm	SEM, TEM, nanoSIMS	Macrophages	Vedhachalam et al. (2007), Nandi et al. (2009), Hafiane and Genest (2017), He et al. (2018)
Migrasomes	0.5–3 µm	SEM, TEM, confocal fluorescence microscopy	NRK cells, MEF, NIH3T3, HaCaT, MGC803, SKOV-3, B16, MDA-MB231, HCT116, SW480	Ma et al. (2015), Huang et al. (2019)
Irregular shaped, crystal-like deposits	<<0.5 µm	STED, AFM, SEM	Macrophages	Jin et al. (2018)
Membrane-derived small vesicles	30–700 nm	SXT, confocal fluorescence microscopy	Primary human fibroblasts	Juhl et al. (2021)
Membrane-derived multivesicular structures	0.5–2.0 µm	SXT, confocal fluorescence microscopy	Primary human fibroblasts	Juhl et al. (2021)
Exosomes	30–150 nm	TEM	Oligondendrocytes, Astrocytes, Primary human fibroblasts	Mutka et al. (2004), Strauss et al. (2010), Ilnytska et al. (2021a)

### 7.4 Ultrastructure of Released Vesicles–do Cells Shed Entire Endo-Lysosomes Containing Cholesterol?

**FIGURE 4:**
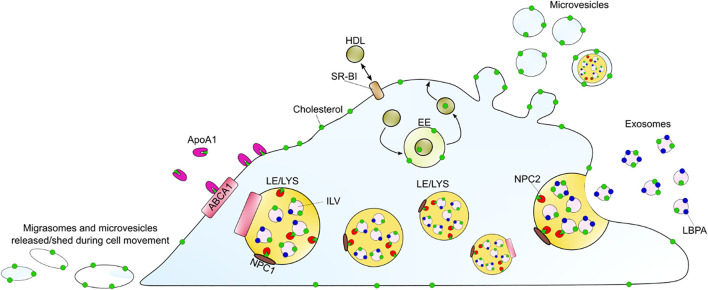
Summary of discussed cholesterol efflux mechanisms. ABCA1 can efflux cholesterol to ApoA1 as the main sterol acceptor. Passive cholesterol exchange between the cell and HDL is mediated by SR-BI. HDL can also gain cholesterol upon endocytosis, passage through endosomes and recycling back to the PM. Additionally, cholesterol might leave the cell by the release of exosomes, upon LE/LYSs fusion with the plasma membrane, or by shedding as microvesicles and/or as migrasomes during cell migration. The LE/LYSs are drawn in different sizes to show the proteins and intraluminal organelles and not to illustrate different populations. ABAC1 (ATP-binding cassette A1), ApoA1 (apolipoprotein A1), EE (early endosomes), HDL (high density lipoprotein), ILV (intraluminal vesicle), LBPA (lysobisphosphatidic acid), LE/LYS (late endosome/lysosome), SR-BI (scavenger receptor BI).

Using a combination of fluorescence and cryo-soft X-ray tomography (cryo-SXT), we have recently shown that human fibroblasts can shed cholesterol-rich microvesicles from the cell surface without chemical pretreatment (Juhl et al., 2021). These vesicles contained several fluorescent cholesterol markers, such as TopFluor-cholesterol or dehydroergosterol, and they could be labeled with filipin. Additionally, some of the microvesicles did contain lysosomal content including NPC2 protein tagged with a red fluorophore and Lysotracker, a content marker for lysosomes (Figure 3A) (Juhl et al., 2021). NPC2 has been shown to be secreted from primary astrocytes independently of secreted cholesterol-rich exosomes, suggesting that the NPC2 containing vesicles we observed in fibroblasts are not exosomes (Mutka et al., 2004; Juhl et al., 2021). Cryo-SXT is a comparably fast 3D ultrastructural microscopy technique, that allows imaging of fully hydrated cryo-fixed cells in less than 1 hour. As an energy source, it typically relies on synchrotron radiation in the energy range between the K-edge absorption of carbon (284 eV) and oxygen (543 eV), also known as the water window (Schneider et al., 2010). Additional staining is not required when imaging biological samples within this water window, since a natural absorption contrast will emerge from the carbon-rich structures. The resolution is down to a few tens of nanometers, and due to the relatively high penetration depth of up to several microns, sectioning of cells is not needed. To obtain 3D tomograms, the sample is tilted around a rotation axis while acquiring single images, that can be aligned and reconstructed (McDermott et al., 2009; Harkiolaki et al., 2018). With cryo-SXT, we were able to resolve the delicate ultrastructure of the shed microvesicles, which revealed internal vesicles in some of them (Figure 3D). Based on these observations of extracellular vesicles containing lysosomal cargo and internal vesicles, we suggest the release of entire LE/LYSs as a new mechanism for cholesterol release (Juhl et al., 2021). This process might be reminiscent of cellular release of melanosomes, which are lysosome-related organelles containing melanin for skin pigmentation (Wu and Hammer, 2014). Transfer of melanosomes from melanocytes to skin keratinocytes involves surface shedding of melanosomes and/or melanosome exocytosis, similar to our observed lysosomal efflux of cholesterol (Wu and Hammer, 2014). Shedding of melanosomes takes place preferentially at sites of surface protrusions, such as filopodia and dendrites, to which melanosomes are reallocated in a Myosin-V and actin-dependent process (Wu and Hammer, 2014). Membrane shedding of entire organelles is likely not related to the formation of apoptotic bodies since it has been observed in other cell types under various conditions, including astrocytes, which were found to release lipid droplets and mitochondria together with ATP (Falchi et al., 2013). Extracellular release of mitochondria could be triggered by CD38-mediated synthesis of the calcium messenger cyclic ADP-ribose in astrocytes and serves a role in protecting neurons after stroke (Hayakawa et al., 2016). Vesicular shedding of lysosome-related organelles has also been identified as a mechanism of drug disposal in brain endothelial cells (Noack et al., 2018). Future studies should be directed towards determining the molecular mechanisms, cells use to shed vesicles containing endo-lysosomes and other organelles, and how such mechanisms could be utilized to secrete lipids, such as cholesterol. A summary of the discussed mechanisms for efflux of cellular cholesterol is shown in Figure 4, below.

## 8 Efflux of Cholesterol Derived From Late Endosomes and Lysosomes

### 8.1 The Role of Niemann Pick Type C Proteins in Export of Cholesterol From Endo-lysosomes

Several organelles can provide cholesterol for efflux, including the cholesterol-rich recycling endosomes, LE/LYSs, the Golgi apparatus, and LDs (Neufeld et al., 2001; Neufeld et al., 2004; Denis et al., 2008; Ouimet et al., 2011; Phillips, 2014). Since most cells receive the majority of their cholesterol from endocytosis of LDL, the export of LDL-cholesterol from endo-lysosomes is closely linked to cholesterol efflux, for example to ABCA1, which resides to some extent in LE/LYSs at steady state (Neufeld et al., 2001). For cholesterol to leave LE/LYSs, the tandem action of the two Niemann Pick type C proteins, NPC1 and NPC2, is crucial. NPC1 is located in the lysosomal membrane and consists of 13 transmembrane helices, containing a sterol-sensing domain, and three luminal domains, a sterol-binding N-terminal domain, a middle luminal domain, and a C-terminal domain (Peake and Vance, 2010; Qian et al., 2020). NPC2 functions in the lumen of the LE/LYSs and is relatively small, consisting of 132 amino acids in its active form, and an additional 19 amino acids signal peptide (Storch and Xu, 2009; Qian et al., 2020). NPC2 binds to and buries the hydrocarbon chain of cholesterol deep into its hydrophobic pocket, in an orientation opposite to that of NPC1 (Infante et al., 2008; Kwon et al., 2009). In 2008, the group of Goldstein and Brown showed that NPC2 was required for the transfer of cholesterol from NPC1 to liposomes, although the transfer mechanism between the two proteins could not be determined from these experiments (Infante et al., 2008). Based on biochemical assays, structural data, and mutagenesis experiments, a model was suggested in which unesterified cholesterol is first bound by NPC2 that hands it over to NPC1 (Infante et al., 2009). Deffieu and Pfeffer, 2011) found that at low pH, NPC2 binds to the second luminal domain of NPC1 and that the strength of the interaction increases in the presence of cholesterol (Deffieu and Pfeffer, 2011). From this study, it was suggested that NPC2 carrying cholesterol would bind to the second luminal loop of NPC1, which would bring the proteins in proximity and mediate the transfer of cholesterol from NPC2 to the N-terminal domain of NPC1. NPC2 would be released from the NPC1, once the latter has accepted the cholesterol, and finally, the cholesterol would be inserted into the membrane of LE/LYSs (Deffieu and Pfeffer, 2011). In support of this model, the crystal structure of an NPC1-NPC2 complex showed that the middle luminal domain of NPC1 binds to the top of NPC2, and revealed a putative cholesterol transfer tunnel between the binding pockets of the two proteins (Li et al., 2016). Moreover, recent studies found an internal tunnel in the structure of yeast and mammalian NPC1 homologs, through which sterols can be transferred to the lysosomal membrane (Winkler et al., 2019; Long et al., 2020). The importance of the NPC proteins is evident from the rare neurogenerative disorder (∼1:120.000 birth) Niemann Pick Type C disease that is caused by lack of functional NPC1 or NPC2 protein. In NPC disease, unesterified cholesterol accumulates in the LE/LYSs together with other lipids such as gangliosides, sphingomyelin, and sphingosine. About 95% of the clinical cases are due to dysfunctional NPC1, however, independent of which protein is disabled, the clinical phenotypes appear similar (Peake and Vance, 2010).

### 8.2 Lipids, NPC Proteins, and ATP-Binding Cassette Transporters Control Cholesterol Efflux From Endo-lysosomes

Vesicles inside the LE/LYSs, known as ILVs, are enriched with cholesterol and lysobisohosphatidic acid (LBPA) (Kobayashi et al., 1999; Möbius et al., 2003; Gruenberg, 2020). LBPA is important for forming the membranes of ILVs and for controlling the cholesterol capacity of LE/LYSs. By treating cells lacking functional NPC1 with LBPA, or treating with its biosynthetic precursor phophatidylglycerol (PG), lysosomal cholesterol clearance was enhanced (McCauliff et al., 2019). Such LBPA/PG mediated cholesterol egress occurrs through increased exosomal secretion (Ilnytska et al., 2021a) and by enhancing the autophagic flux in cells lacking functional NPC1 (Ilnytska et al., 2021b). Interestingly, LBPA is not able to reduce cholesterol levels in cells expressing NPC2 protein mutated in the hydrophobic knob domain, which has been shown to directly interact with LBPA (McCauliff et al., 2019). Additionally, the efficiency of NPC2 to transfer cholesterol between membranes was found to be enhanced in the presence of LBPA, and inhibited by anti-LBPA antibodies (Xu et al., 2008). It is likely that NPC2 shuttles cholesterol from ILV membranes to the limiting LE/LYS membrane, making it available for other sterol transfer proteins (Storch and Xu, 2009). Dysfunction of NPC1, the cholesterol export protein in the endo-lysosomal limiting membrane, but not the absence of NPC2, can be rescued by overexpression of ABCA1 (Choi et al., 2003; Boadu et al., 2012). These important observations suggest that NPC2 is needed to solubilize cholesterol inside of the lysosome and donate it to different transporters including NPC1 and ABCA1 for export from this compartment. In line with this conclusion is our recent observation that treating NPC2-deficient human fibroblasts with purified NPC2 mobilized cholesterol from endo-lysosomes towards the PM, which was paralleled by reallocation of endo-lysosomes to the periphery and direct sterol transfer to the PM (Juhl et al., 2021). The latter could be shown by bleaching TopFluor-cholesterol in a portion of the PM and measuring a decrease in fluorescence of this sterol probe in nearby LE/LYSs underneath the PM (Juhl et al., 2021). Using kinetic modeling of such a fluorescence loss in photobleaching (FLIP) experiment, we inferred a dynamic pool of sterol in endo-lysosomes in exchange with the PM, which has a residence time in LE/LYSs of about 40 s (Juhl et al., 2021). Subsequent cholesterol efflux from the PM was independent of NPC2 but stimulated by LXR agonists and apoA1, suggesting that both pathways work in tandem (Juhl et al., 2021). Reallocation of endo-lysosomes to the cell periphery and reduction of the cholesterol storage phenotype was also found in fibroblasts lacking functional NPC1 upon treatment with PG (Ilnytska et al., 2021a). Some of those LE/LYSs might release their cholesterol content as exosomes, i.e., by lysosomal exocytosis, while vesicular shedding of lysosomal cargo from the PM could contribute as well. Interestingly, macrophages from NPC1 knockout (NPC1^−/−^) mice have reduced cholesterol efflux capacity, which could be overcome by stimulating ABCA1 transporters with LXR agonists. Heterozygous NPC1^+/−^ mice show less apoptosis and lesional necrosis, likely because cholesterol transport from lysosomes to the ER is impaired, as this pathway is essential for inflammosome activation and the UPR stress response (Feng et al., 2003; De la Roche et al., 2018). Similarly, incorporation of fatty acids derived from atherogenic LDL into phospholipids is impaired in NPC1-deficient macrophages (Leventhal et al., 2004). Together, these studies suggest that NPC1 and NPC2 provide cholesterol and phospholipids from LE/LYSs for efflux from cells.

### 8.3 The Connection Between Endo-lysosomes and Lipid Droplets in Cellular Cholesterol Efflux

To understand the role of lysosomes in the trafficking of lipoprotein-derived cholesterol, it is important to realize that cellular efflux of LDL-derived cholesterol differs between native LDL and oxidized, acetylated, or aggregated LDL, as such atherogenic LDL is differently processed in endo-lysosomes (Dhaliwal and Steinbrecher, 2000; Wang M.-D. et al., 2007; Haka et al., 2009). For example, aggregated and matrix-retained LDL in the intima of the vessel wall gets attacked by macrophages, which attempt to engulf the aggregated LDL, thereby triggering secretion of hydrolytic enzymes including acid lipase from lysosomes (Haka et al., 2009). This, in turn, hydrolyzes CEs residing in aggregated LDL. To prevent a collapse of the pH gradient needed for optimal function of these enzymes, macrophages secrete lysosomal acid lipase into the contact area, thereby forming a tightly sealed so-called “lysosomal synapse” allowing for extracellular degradation of LDL’s sterol esters (Singh et al., 2016). Upon uptake into the cells, the excess cholesterol is re-esterified and stored in LDs, whose abundant appearance converts those macrophages into foam cells. Thus, stored CEs in LDs represent another major intracellular source of cholesterol for efflux (Ouimet et al., 2011). CEs can be re-hydrolyzed for sterol mobilization via cytoplasmic cholesteryl ester hydrolase (Brown et al., 1979). Ingestion of LDs into lysosomes, so-called lipophagy, and hydrolysis of stored CEs by acid lipase provides significant amounts of cholesterol for efflux from macrophage foam cells (Ouimet et al., 2011; Robichaud et al., 2021). Lipophagy and likely other forms of autophagy are impaired in mammalian cells, lacking functional NPC1 or NPC2, suggesting that these sterol transporters play an important role in the efflux of droplet-derived sterols from lysosomes (Sarkar et al., 2013; Guo et al., 2016; Robichaud et al., 2021). This is likely an evolutionary ancient function of these proteins since impaired lipophagy is also observed in yeast Saccharomyces cerevisiae lacking functional NPC homologs, NCR1 or Npc2 (Tsuji et al., 2017; Winkler et al., 2019). Since these cells lack a lipoprotein pathway, yeast relies on sterol mobilization from LDs in the vacuole during starvation.

## 9 Formation and Efflux of Oxysterols

### 9.1 Oxysterols as Polar Derivatives of Cholesterol Which Regulate Sterol Homeostasis and Efflux

Oxysterols are oxidized derivatives of cholesterol bearing additional hydroxy-, keto- or epoxy groups which significantly increase their water solubility compared to the parent cholesterol molecule (Luu et al., 2016). Since increased polarity results in lower membrane retention and higher inter-membrane transfer, oxysterols move very fast through cells and are more efficiently removed from cells compared to cholesterol (Sinensky, 1981; Lange et al., 1995; Morel et al., 1996). For example, the transfer from red blood cells to plasma lipoproteins is about 2000-fold faster for 25-hydroxycholesterol than for cholesterol (Lange et al., 1995). Sidechain oxidized cholesterol derivatives, such as 25-or 27-hydroxycholesterol, can form from LDL-derived cholesterol upon export from endo-lysosomes and transport to the ER or mitochondria, respectively (Axelson and Larsson, 1995; Frolov et al., 2003). Export of these oxysterols from endo-lysosomes depends on NPC1, and to a lower extent on NPC2 (Frolov et al., 2003; Abi-Mosleh et al., 2009; Petersen et al., 2020). 25- hydroxycholesterol can activate LXRs to stimulate ABCA1 mediated cholesterol efflux, and this oxysterol is itself efficiently removed from cells by ABCA1 (Venkateswaran et al., 2000; Tam et al., 2006). Therefore, impaired oxysterol formation in NPC1-deficient cells will likely contribute to the reduced efflux capacity and reduced HDL formation in NPC1 disease (Choi et al., 2003). The same is probably the case for Wolman disease and cholesteryl ester hydrolase deficiency, which both lead to accumulation of CEs in endo-lysosomes due to defective or absent acid lipase and thereby impaired ABCA1 mediated cholesterol efflux (Bowden et al., 2011). NPC2 binds 25-hydroxycholesterol with a much lower affinity than cholesterol and is probably less important for the export of oxysterols from endo-lysosomes because the water solubility of 25- and 27-hydroxycholesterol is sufficient for spontaneous transfer (Frolov et al., 2003; Petersen et al., 2020). Sidechain oxidized cholesterol derivatives move rapidly across membranes and can increase the permeability of lipid membranes to small molecules and ions, which could contribute to lysosome destabilization by oxysterols (Yuan et al., 2000; Kulig et al., 2018). In parallel, these oxysterols cannot order membranes as much as cholesterol does, and they might increase the propensity of cholesterol to partially protrude from the bilayer, thereby increasing the pool size of “accessible” cholesterol for efflux to acceptors (Bielska et al., 2014). However, there is also evidence that oxysterols, such as 25-hydroxycholesterol, actually lower cellular cholesterol efflux capacity (Kilsdonk E. P. et al., 1995; Luu et al., 2016). This could be due to stimulation of ACAT resulting in cholesterol esterification and storage in LDs.

### 9.2 Analysis of Oxysterol Trafficking and Efflux by Fluorescence Microscopy

**FIGURE 5:**
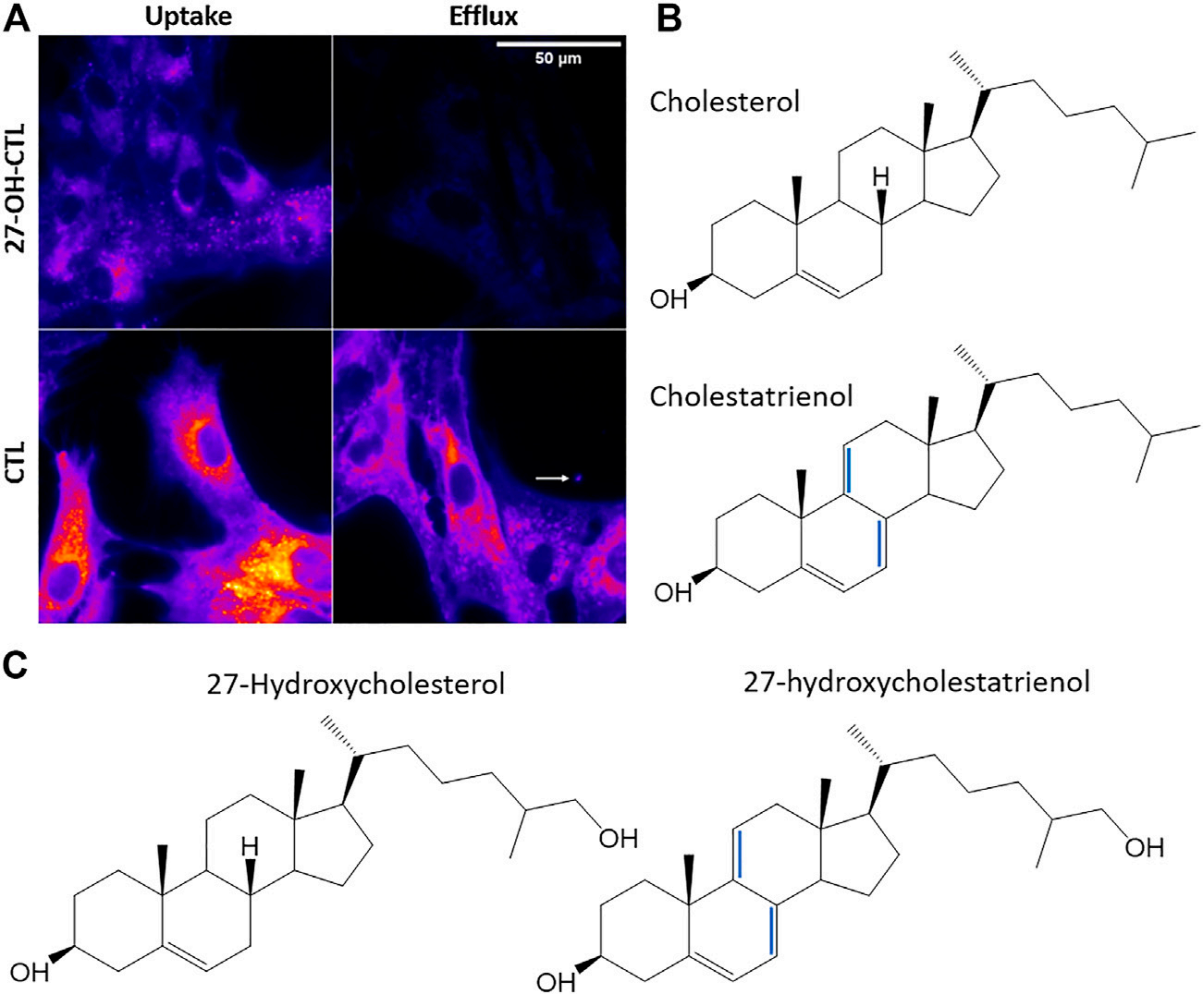
Uptake and efflux of fluorescent oxysterols in human fibroblasts. **(A)** uptake and efflux of cholestatrienol (CTL) and 27-hydroxycholestatrienol (27-OH-CTL) in human fibroblasts studied by UV-sensitive fluorescence microscopy. **(B)** structures of cholesterol and its fluorescent analogue CTL. **(C)** 27-hydroxycholesterol and its fluorescent analogue 27-OH-CTL. Extra double bonds in the fluorescent analogs are the only modifications compared to the natural sterols and are shown in blue. Figure adapted and reproduced from (Szomek et al., 2020) with permission.

The lack of appropriate tools to follow oxysterol transport in living cells has limited our knowledge about intracellular trafficking of oxysterols. Given that oxysterols, such as 25- or 27-hydroxycholesterol, differ from cholesterol only by having one additional hydroxy group, additional modifications to introduce fluorescent moieties for live-cell imaging must be kept to an absolute minimum. One approach is two add an alkyne group to the oxysterol, load this analogue into cells and covalently link it to a fluorescent group by click chemistry (Nedelcu et al., 2013; Peyrot et al., 2014). This approach allows for high-resolution imaging of oxysterol distribution, but it cannot be ruled out that either the click reaction or the attached dye molecule have an impact on this distribution. Also, imaging of the fluorescent construct is not possible in living cells. In a pioneering study, Iaea et al. (2015) presented a novel intrinsically fluorescent analogue of 25-hydroxycholesterol, which contains only two additional double bonds in the ring system compared to the parent oxysterol (Iaea et al., 2015). This analogue named 25-hydroxycholestatrienol can suppress cholesterol synthesis, activate ACAT for sterol esterification, be esterified in cells and stimulate expression of ABCA1 via activation of LXRs (Iaea et al., 2015). Its export from lysosomes depends on NPC1 and to a lower extent on NPC2 (Iaea et al., 2015; Petersen et al., 2020). Based on the same design strategy, we have recently developed a novel derivative of 27-hydroxycholesterol, 27-hydroxycholestatrienol (27-OH-CTL), which differs from 27-hydroxycholesterol only by two additional double bonds in the steroid ring system (Figure 5), allowing for spectroscopic analysis and live-cell imaging by ultraviolet-sensitive microscopy, similar to 25-hydroxycholestatrienol (Szomek et al., 2020). We compared the intracellular trafficking of 27-OH-CTL with that of cholestatrienol (CTL), which has identical photophysical properties and just two additional double bonds compared to cholesterol (Figure 5). Thus, the difference between 27-OH-CTL and CTL is only one extra hydroxy group at carbon 27, exactly as the difference between 27-hydroxycholesterol and cholesterol (Figure 5). This enabled us to study the effect of one additional hydroxy group in the steroid side chain on intracellular sterol trafficking and efflux. We found that 27-OH-CTL and CTL show a similar extent of non-vesicular transport, as measured by fluorescence recovery after photobleaching (not shown but see Szomek et al. (2020)). However, 27-OH-CTL was much more efficiently effluxed from cells than CTL (Figure 5A) and 27-OH-CTL but not CTL accumulated a lot in LDs in human fibroblasts (Szomek et al., 2020). Comparable intracellular transport kinetics but more rapid and extensive efflux, as well as the metabolic conversion, was also found for 25-hydroxycholesterol compared to cholesterol in macrophages using radioactive sterol probes (Morel et al., 1996). Thus, cells can sensitively adapt transport and efflux to even minor structural changes of sterol molecules, and these processes can be studied using radioactive tracers but also directly observed in cells using intrinsically fluorescent analogues.

## 10 Conclusion and Outlook

Since mammalian cells cannot degrade cholesterol, they have developed intricate mechanisms for its disposal. Here, we have summarized our current understanding of the cellular mechanisms of cholesterol efflux. A particular challenge is the extremely low water solubility of cholesterol, which cells overcome by employing several mechanisms. In this review we discussed in detail; 1) how cells use a variety of transporters to shuttle cholesterol between membrane and lipoproteins; 2) how cells generate close protein-mediated membrane contacts to accelerate sterol exchange and 3) how they couple cholesterol export to the formation of extracellular carrier vesicles and lipoproteins. In addition to the classical pathways of reverse cholesterol transport and formation of HDL, we highlighted novel findings of the concerted action and molecular function of a variety of ABC transporters. We also provided an overview of recently described vesicular secretion pathways for cholesterol, ranging from the secretion of exosomes over shedding of ectosomes from the PM to the formation of migrasomes, and we explained, how those mechanisms might contribute to net cholesterol efflux from cells. A particular focus was set on human diseases related to disturbed cholesterol efflux, either at the cell surface or due to defective mobilization from intracellular sites, such as endo-lysosomes and LDs. Finally, we explained the important link between cholesterol efflux and the formation and export of oxysterols from cells. However, many questions remain and future studies could focus on the following:

• What is the nature of active cholesterol, and how is the cholesterol pool accessible to bacterial toxins related to cholesterol efflux and precipitation from membranes? While being a powerful concept, the molecular picture of “active cholesterol” is very vague at the moment. New techniques, including novel biophysical and imaging approaches, are required to substantiate this concept in living cells. Also, a better understanding of the molecular mechanisms underlying the binding of bacterial toxins to cellular membranes is needed to ensure that the observed threshold phenomena indeed solely reflect different cholesterol pools in the membrane.
• Are there other mechanisms of controlling the chemical potential of cholesterol and thereby its fugacity in cellular membranes and lipoproteins? Our current understanding of the mechanisms regulating cholesterol’s chemical potential in cellular membranes and lipoproteins are based on a large body of physico-chemical data about specific phospholipid-cholesterol interactions in model membrane systems. Such systems are necessarily much simpler than cellular membranes, and we might miss an important piece of the puzzle by relying too much on model membrane studies. Thus, more biophysical studies in living cells and development of more realistic model systems are needed to answer this question.
• How important are membrane contact sites for cellular efflux of cholesterol? It is well known that close, protein-mediated contact between organelles are involved in intracellular cholesterol transport, but it is little understood about whether the organelle interactome also regulates cellular cholesterol efflux and how sterol transporters, such as GramD1/Aster or oxysterol binding proteins function at those sites during export of excess cholesterol from cells.
• What are the molecular mechanisms underlying shedding of cholesterol-rich vesicles from the PM? Secretion of micro-vesicles as an export mode for cholesterol from cells is an exciting new field that will complement and expand our understanding of cholesterol efflux in the future. Understanding the underlying mechanisms of vesicle secretion and their regulation will be possible by combining live-cell and correlative super-resolution microscopy with genetic knockdown techniques in different cell types and under various cholesterol loading conditions.
• How is the recruitment of endo-lysosomes to the PM for cholesterol efflux regulated at a molecular level? Exciting new findings have revealed the importance of intracellular location and dynamics for lysosome function, and lysosomal cholesterol appears to be an important regulating factor in this context. Reallocation of LE/LYSs to the PM has been found to be linked to cholesterol efflux, and determining the underlying molecular mechanisms will enable us to understand, how intracellular cholesterol is mobilized for efflux from cells.
• Is there a quantitative contribution of the release of entire LE/LYSs to net cholesterol efflux, and if so, what are the underlying mechanisms? Shedding of vesicles containing entire endo-lysosomes and other organelles has been observed during cholesterol efflux, but also in other cellular processes and various cell types. Whether this indeed plays a physiological role or is exaggerated under cell culture conditions needs to be explored in the future.
• What are the molecular mechanisms and biophysical principles of oxysterol transport and efflux from cells and how can oxysterols control cholesterol efflux at the cellular and molecular level? Oxysterols play an important role in cholesterol efflux; they are efficiently exported from cells due to their higher water solubility compared to cholesterol, and they regulate cellular cholesterol efflux as ligands of sterol-metabolizing enzymes and transcription factors. Future studies could combine novel imaging technologies with analytical approaches, such as mass spectrometry to study trafficking and metabolism of oxysterols at a cellular and tissue level.

In the past, mechanisms of cellular cholesterol efflux were explored primarily by biochemical approaches, and this has generated a large amount of data on the formation and clearance of HDL. With the increasing use of quantitative live-cell imaging, super-resolution microscopy and correlative imaging, elucidation of the spatiotemporal orchestration of cholesterol efflux at a subcellular level becomes possible. We expect this trend to continue in the future with novel imaging technologies further contributing to our understanding of this important process.
